# *WaySafe*: improving decisionmaking around health risk behaviors for prisoners transitioning back to the community

**DOI:** 10.1186/1940-0640-10-S1-A55

**Published:** 2015-02-20

**Authors:** Grace A Rowan, Wayne EK Lehman, George W Joe, Kevin Knight, Yang Yang

**Affiliations:** 1 Institute of Behavioral Research, Texas Christian University, Fort Worth, TX, 76129, USA; 2 Department of Psychology, University of Louisiana at Lafayette, Lafayette, LA, 70504, USA

## 

Many individuals enter the U.S. prison system with a history of high-risk drug and sexual practices, which are two primary contributors to the high rates of HIV and hepatitis cases among this population. As these individuals return to the community, they are likely to continue high-risk behaviors, making it critical that decisionmaking programs designed to reduce risky behaviors be instituted during incarceration close to their release. Prior research on Texas Christian University (TCU) Mapping-Enhanced Counseling (included in the National Registry of Evidence-based Programs and Practices as an evidence-based practice) has identified its utility for improving decisionmaking, communication, problem exploration, and personal planning through the use of graphical representations designed to help clients “see” the links and relationships among thoughts, ideas, feelings, and behaviors. It serves as an important tool for helping move traditional, public health-focused educational efforts toward a more comprehensive approach that addresses the challenges of personal decisionmaking around sex and drug use behaviors.

The TCU Disease Risk Reduction (DRR-1) project developed and tested an intervention, *WaySafe*, designed to increase positive decisionmaking skills among offenders for healthy living, including skills for reducing disease risk behaviors. *WaySafe* uses TCU Mapping-Enhanced Counseling to focus on the cognitive aspects of risky sexual and drug use behaviors during re-entry to improve problem recognition, commitment to change, and strategies for avoiding behavioral risks of infections. *WaySafe* includes six 1-hour, highly interactive sessions to increase motivation and planning. A total of 1398 incarcerated offenders from eight different correctional facilities in two states participated in the study during the last phase of their in-prison substance abuse treatment prior to transition back to the community.

All participating offenders completed baseline surveys and then were randomly assigned to the *WaySafe* intervention or treatment as usual (TAU). At the completion of the 6-week *WaySafe* curriculum, participating offenders completed follow-up surveys. Baseline and follow-up surveys included measures of knowledge, confidence, and motivation regarding general HIV information, risky sex and drug use, what to do if exposed, and general life skills. Results supported hypotheses that offenders in *WaySafe* improved significantly more than TAU clients on knowledge, attitudes, and behavioral intentions to avoid risks. Finding new methods to reinforce the lessons learned in the community is a critical next step, because these changes in attitudes and intentions will degrade over time with the stress and pressures of transitioning from prison to the community. Reinforcing lessons learned while offenders are currently facing risky situations may be helpful.

## Trial registration

Clinicaltrials.gov NCT01900210

